# New Inhibitor Based on Hydrolyzed Keratin Peptides for Stainless Steel Corrosion in Physiological Serum: An Electrochemical and Thermodynamic Study

**DOI:** 10.3390/polym16050669

**Published:** 2024-02-29

**Authors:** Adriana Samide, Gabriela Eugenia Iacobescu, Bogdan Tutunaru, Cristian Tigae, Cezar Ionuţ Spînu, Bogdan Oprea

**Affiliations:** 1 Department of Chemistry, Faculty of Sciences, University of Craiova, 107i Calea Bucuresti, 200478 Craiova, Romania; cezar.spinu@edu.ucv.ro; 2 Department of Physics, Faculty of Sciences, University of Craiova, 13 A. I. Cuza, 200585 Craiova, Romania; gabriela.iacobescu@edu.ucv.ro; 3 Faculty of Medicine, University of Medicine and Pharmacy, Petru Rares, 2, 200349 Craiova, Romania

**Keywords:** stainless steel, corrosion inhibition, hydrolyzed keratin peptides, electrochemical and thermodynamic study, AFM investigation

## Abstract

Reducing the impact of some biological fluids on bioimplants involves the control of surface characteristics by modeling the interface architecture and assembling ecofriendly thin films to retard corrosion. Therefore, a mixture of hydrolyzed keratin peptides (HKER) was investigated as a corrosion inhibitor for 304L stainless steel (SS) in physiological serum (PS), using electrochemical measurements associated with optical microscopy and atomic force microscopy (AFM). The tests, performed for various concentrations of the inhibitor at different temperatures, showed that the inhibition efficiency (*IE*) decreased with a rise in temperature and proportionally increased with the HKER concentration, reaching its maximum level, around 88%, at 25 °C, with a concentration of 40 g L^−1^ HKER in physiological serum. The experimental data best fitted the El-Awady adsorption model. The activation parameters (E_a_, ${∆H}_{a}$ and ${∆S}_{a}$) and the adsorption ones (${∆G}_{ads}^{0}$, ${∆H}_{ads},$ ${∆S}_{ads}$) have highlighted a mixed action mechanism of HKER, revealing that physisorption prevails over chemisorption. AFM parameters, such as the average roughness (*R_a_*), root-mean-square roughness (*R_q_*) and maximum peak-to-valley height (*R_p−v_*), confirmed HKER adsorption, indicating that a smoother surface of the 304L stainless steel was obtained when immersed in a PS-containing inhibitor, compared to the surface designed in blank solution, due to the development of a protective layer on the alloy surface.

## 1. Introduction

Numerous ways of reducing corrosion effects that induce changes in the characteristics and architecture of metal surfaces have been approached. Thus, the assembly of some protective coatings at the metal/environment interface, through the adsorption of natural or synthetic polymers on the metal surface, leads to the delay/blocking of corrosive processes. The polymer inhibitor action mechanism consists of the physical, chemical or mixed adsorption (covering both features) of macromolecules possessing numerous active centers. These determine the polymer’s ability to interact with the metal surface, forming protective films and, thus, blocking the effects of metallic oxidation and corrosion processes, respectively [1,2,3].

The polymer inhibition efficiency depends on the coating uniformity and stability conferred by its nucleophilic character, involving the macromolecule’s ability to donate electrons, the metal surface morphology, environmental composition and pH [1,4].

Therefore, a particular attention has been paid to the corrosion inhibition processes of metals/alloys with protective films, developed by the adsorption of some polymers on their surface. The anticorrosion performance of various materials, including polyvinyl acetate [5], polyvinyl alcohol/silver nanoparticle composite [6,7], their copolymers, such as poly(vinyl butyral-co-vinyl alcohol-co-vinyl acetate) [8], polyamide [9], polypyrrole [10], chitosan [11] and polyacrylic acid [12], was reported for the corrosion inhibition of copper in different media [9,10,11], carbon steel [5,8], stainless steel [6,7] and aluminum [12]. Additionally, self-healing vinyl-ester polymer [13] and epoxy [14] based coatings, as well as *Prunus domestica* gum-grafted polyaniline (PDG-g-PANI) composite [15], were used to promote carbon steel [13,14,15] and stainless steel [15] corrosion inhibition.

Proteins derived from various sources have also been studied as corrosion inhibitors. Bovine serum albumin [16] and bovine cheese protein [17], as well as corn gluten powder [18], were investigated as corrosion inhibitors for magnesium alloy [16] and steel [17,18].

Small peptides have also been studied as potential corrosion inhibitors for different metal substrate [19,20,21]. Theoretical studies using computational methods such as density functional theory (DFT) and Monte Carlo (MC) revealed that small peptides, such as L-alanine dipeptide and tripeptide, have a stronger adsorption ability on the iron surface than on copper and aluminum. This makes them a good proposal as corrosion inhibitors in perspective [19]. A mixture constituted from dipeptide of glycine and glutamic acid (Gly-Glu), containing cysteine (Gly-Glu + Cys) and tripeptide of glycine, cysteine and glutamic acid (glutathione), were reported as effective inhibitors for carbon steel corrosion using experimental and theoretical studies [20].

Keratin composition consists of a wide range of water-insoluble fibrous proteins. Hard keratins containing a high cysteine residue and disulfide bonds, originating from hair and nails or wool, horns, hooves, chicken feather, claws [22,23,24,25] and other similar animal tissues, are employed in cosmetics more than soft cytoplasmic epithelial keratins that link skin cells together, protecting them from degradation caused by the environment [22]. Three classes of keratin proteins extracted from hair were reported, including α-Keratin, with an average molecular weight of 60–80 kDa and low sulfur concentrations, found in the hair fiber cortex; β-Keratin, from hair cuticles, and γ-Keratin, with a molecular weight of around 15 kDa, and a high amount of sulfur [23].

Chemical hydrolysis, enzymatic and microbial treatment, dissolution in ionic liquids, microwave irradiation and thermal hydrolysis or a superheated process were used to prepare hydrolyzed keratin, consisting of small molecular peptides containing amino acids, such as alanine, arginine, aspartic acid, cysteic acid, cysteine, glutamic acid, glycine, histidine, isoleucine, lanthionine, lysine, leucine, methionine, proline, phenylalanine, serine, threonine, tyrosine, valine [25].

Stainless steels are commonly employed in the manufacture of industrial equipment functioning in aggressive environments, such as bioimplant and food packaging materials, as well as construction materials in the chemical, dairy and beverage industry and wastewater treatment [26]. The stainless steel corrosion resistance is due to the occurrence of a protective layer, with a thickness of 1–4 nm on its surface [26].

In this study, the corrosion inhibition of 304L stainless steel (SS) in physiological serum (PS) containing a hydrolyzed keratin (HKER) was investigated using electrochemical measurements, such as open circuit potential (OCP), electrochemical impedance spectroscopy (EIS), potentiodynamic polarization (PP) assisted by optical microscopy and atomic force microscopy (AFM).

Hydrolyzed keratin peptides are water-soluble and toxicity-free. In addition, the peptide molecular group provides additional adsorption centers, interacting with the metal surface by donor–acceptor processes, forming a protective film that improves substrate corrosion inhibition.

Consequently, an optimal application of the hydrolyzed keratin peptides (HKER) synthesized from sheep wool by a combined chemical–physical procedure was designed, integrating the peptide mixture among other analogues reported as effective corrosion inhibitors. The comprehensive approach and the outcomes provided by this study contribute to the elucidation of the action mechanism of a mixture of peptides that interact with stainless steel, controlling its surface deterioration.

## 2. Materials and Methods

### 2.1. Materials

The 304L stainless steel (SS), an alloy of type FeCr18Ni10 obtained from Merck (Darmstadt, Germany) contains (wt %): C ≤ 0.03%; Cr—18–20%; Ni—8–12%; Mn—2.0%, with Fe in balance. This is the typical composition of 304L stainless steel [27,28].

Analytical-purity NaCl from Merck (Darmstadt, Germany) and bi-distilled water were used to prepare the aggressive environments inducing corrosion on 304L stainless steel (SS), including the following: (1) physiological serum (PS) consisting of 0.9% NaCl aqueous solution, hereinafter referred to SS/PS; (2) physiological serum containing different concentrations of HKER, namely: 10 mg L^−1^; 15 mg L^−1^; 20 mg L^−1^; 25 mg L^−1^; 30 mg L^−1^; 35 mg L^−1^; 40 mg L^−1^. Therefore, the corrosion systems will be further named as follows: SS/PS/10 mg L^−1^ HKER; SS/PS/15 mg L^−1^ HKER; SS/PS/20 mg L^−1^ HKER; SS/PS/25 mg L^−1^ HKER; SS/PS/30 mg L^−1^ HKER; SS/PS/35 mg L^−1^ HKER; SS/PS/40 mg L^−1^ HKER.

The hydrolyzed keratin peptides (HKER), synthesized at The National Research & Development Institute for Textiles and Leather, Bucharest, Romania, from sheep wool by alkaline hydrolysis [29], are a water-soluble cream-colored powder consisting of the following: hydrolyzed keratin peptides, with small molecular weight (max 2285 Da), and cysteine residues, characterized by the presence of amine nitrogen and cystine sulfur. The content of amino acids in peptides is similar to that of hair, consisting of cysteine, glutamine, lysine, arginine, histidine valine, alanine, leucine, proline, isoleucine, threonine, methionine, phenylalanine, tyrosine, asparagine, etc.

### 2.2. Methods

#### 2.2.1. Electrochemical Measurements

Both electrochemical impedance spectroscopy (EIS) and potentiodynamic polarization were performed, using a standard electrochemical cell with three electrodes coupled to a potentiostat/galvanostat VoltaLab 40 (Radiometer Analytical SAS, Lyon, France) with VoltaMaster 4 software (version 7.8.26338.3) (corrosion option), which allows the simultaneous evaluation of corrosion current density and corrosion rate (potentio-gravimetric method). The software specification indicates that the standards for electrochemical measurements are the ASTM-G3 [30] and ASTM-G5 [31] and CNC ASTM corrosion cell guidelines.

The working electrode was composed of a 304L stainless steel plate with an area of 1.0 cm^2^; the auxiliary electrode was made from a platinum plate (with an area of 1.0 cm^2^). The Ag/AgCl electrode was used as a reference. Before testing, the stainless steel plates were mechanically polished with emery paper, ultrasonically cleaned, degreased with acetone and dried in warm air. Additionally, a thermostatic heating device was used. The environment temperature from the electrochemical cell was continuously controlled with a specific thermometer. The electrochemical assembly was also reported in our previous studies [5,6,7,8,32].

##### Electrochemical Impedance Spectroscopy (EIS)

The electrochemical impedance spectroscopy analysis was carried out, at open circuit potential, after ten minutes from immersing the electrodes in the corrosive environment, in the frequency range of 10^5^ Hz and 10^−1^ Hz, with an AC perturbation signal of 10 mV. The Nyquist and Bode diagrams were recorded at temperatures of 25 °C and 45 °C, respectively, for 304L stainless steel (SS) immersed in physiological serum blank solution (SS/PS) and in physiological serum containing 30 mg L^−1^ inhibitor (SS/PS/30 mg L^−1^ HKER) and, respectively, 40 mg L^−1^ (SS/PS/40 mg L^−1^ HKER).

##### Potentiodynamic Polarization (PDP)

The potentiodynamic polarization analysis was carried out after EIS for the same solutions and temperatures, with a potential scan rate of 1.0 mV s^−1^, in a potential range from −1.0 V to 1.0 V. The semi-logarithmic potentiodynamic curves were recorded across the entire potential range. The Tafel diagrams were processed in the interest area where the steel is active, namely, in the potential range of ±200 mV with respect to the corrosion potential values. In addition, to calculate the thermodynamic parameters of activation and adsorption, the corrosion rates were determined using the potentio-gravimetric method, in the potential range where the stainless steel is active, mentioned above, at four temperatures: 25 °C, 35 °C, 45 °C and 55 °C. SS was successively immersed in PS blank solution and PS containing the following HKER concentrations: 10 mg L^−1^, 15 mg L^−1^, 20 mg L^−1^, 25 mg L^−1^, 30 mg L^−1^, 35 mg L^−1^ and 40 mg L^−1^, further denoted: SS/PS (blank solution); SS/PS/10 mg L^−1^ HKER; SS/PS/15 mg L^−1^ HKER; SS/PS/20 mg L^−1^ HKER; SS/PS/25 mg L^−1^ HKER; SS/PS/30 mg L^−1^ HKER; SS/PS/35 mg L^−1^ HKER; SS/PS/40 mg L^−1^ HKER. Four sets of samples were prepared (one set for each temperature); each set contained eight plates made from 304L stainless steel, with an active area of 1.0 cm^2^; the stages of processing, cleaning and drying were carried out; for each sample, the temperature was fixed in the corrosive environment; after 10 min of immersion, linear potentiometry with the potential scan rate of 1.0 mV s^−1^. This was applied within a restricted potential range of ±500 mV with respect to the OCP; the potentiodynamic curves were processed as semi-logarithmic curves within the potential range of ± 200 mV on both sides of the corrosion potential; the software option for the calculation of corrosion parameters was accessed, and the corrosion rate (*CR*), expressed in μm year^−1^, was revealed.

#### 2.2.2. Surface Characterization

Optical microscopy and atomic force microscopy (AFM) were used to examine the 304L stainless steel surface morphology before and after corrosion in PS, both with and without HKER.

##### Optical Microscopy

The optical microscopy images were acquired for the 304L stainless steel samples before corrosion (control sample—standard) and after the potentiodynamic polarization performed at 25 °C and 45 °C, respectively, as follows: SS corroded in PS without inhibitor; SS corroded in PS containing 30 mg L^−1^ HKER; SS corroded in PS containing 40 mg L^−1^ HKER. A metallographic microscope, EUROMEX, with a Canon camera and the included software was used, as reported in our previous studies [7]. The following characteristics were used to collect all the examined images: eyepiece magnification power: 10; lens magnification power: 40; magnifying power of the microscope: 400.

##### Atomic Force Microscopy (AFM)

The surface morphology of the 304L stainless steel before and after electrochemical measurements, performed at 25 °C in PS both in the absence and presence of 30 mg L^−1^ and 40 mg L^−1^ HKER, were observed by non-contact mode atomic force microscopy, using the NC-AFM, PARK XE-100 SPM system. The cantilever had a nominal length of 125 mm, a nominal force constant of 40 N/m, and oscillation frequencies in the range of 275–373 kHz. The average roughness (*R_a_*), root-mean-square roughness (*R_q_*) and maximum peak-to-valley height (*R_p−v_*) of the surfaces were estimated over the areas of 45 × 45 μm^2^. The devices were also described in our early studies [6,8].

## 3. Results and Discussion

### 3.1. Open Circuit Potential (OCP)

**Figure 1:**
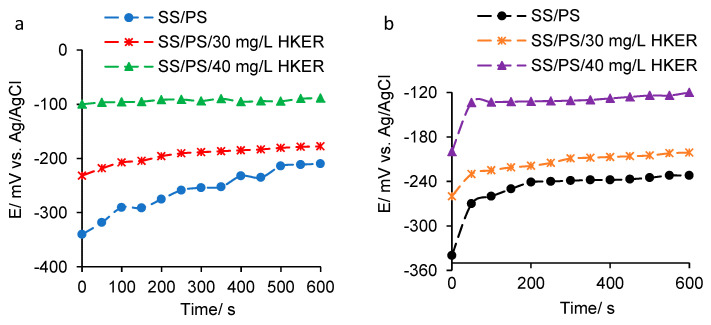
The OCP diagrams of 304L stainless steel in physiological serum, in the presence and absence of HKER: (**a**) at 25 °C; (**b**) at 45 °C.

The open circuit potential variation for 304L stainless steel immersed in physiological serum, both in the absence and presence of HKER, at 25 °C and, respectively, 45 °C is presented in Figure 1.

In the HKER presence, the potential moves in the positive direction, as its concentration increases, probably due to spontaneous adsorption of HKER molecules on the alloy surface. Therefore, a protective coating that interposes at the metal/environment interface, thus improving the system equilibrium, was formed, with the ionic transfer from the metal surface to the electrolyte and vice versa being delayed.

At 45 °C, in the presence of HKER, the potential stabilizes at more negative values compared to those recorded at 25 °C (Table 1), probably due to the fact that a higher temperature slightly facilitates the desorption process of HKER molecules. In the absence of HKER, at a temperature of 45 °C, the diffusion rate of the chemical species from the solution to the electrode surface increases compared to that at 25 °C, affecting more strongly the balance in the electric double layer and leading to an increase in the Cl^−^ concentration at the steel/electrolyte interface. Therefore, the negative charge at the surface level is higher, inducing a displacement of the potential in the open circuit to a more negative value compared to that recorded at 25 °C.

### 3.2. Electrochemical Impedance Spectroscopy (EIS)

**Figure 2:**
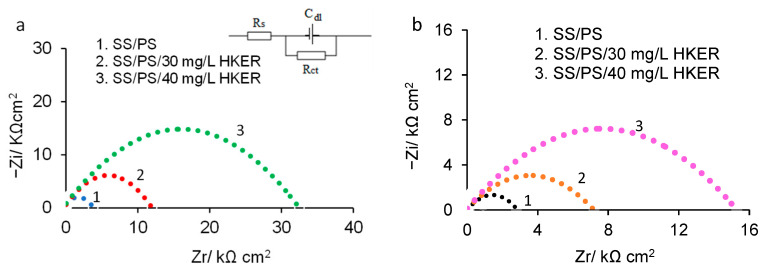
Nyquist plots recorded for 304L stainless steel immersed in physiological serum, both in the presence and absence of HKER: (**a**) at 25 °C; (**b**) at 45 °C.

**Figure 3:**
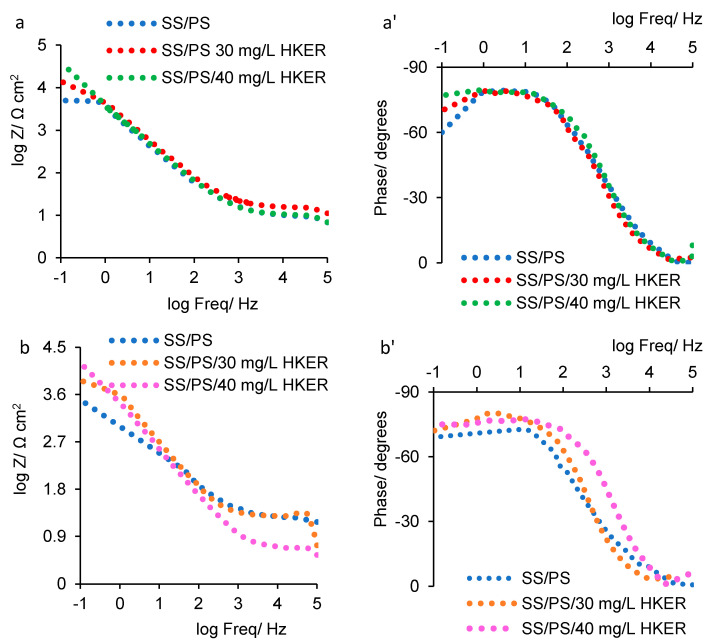
Impedance Bode diagrams (**a**,**b**) and phase Bode diagrams (**a’**,**b’**) recorded for 304L stainless steel immersed in physiological serum, both in the presence and absence of HKER: (**a**,**a’**) at 25 °C; (**b**,**b’**) at 45 °C.

Figure 2 and Figure 3 display the Nyquist and Bode diagrams recorded in the frequency range from 10^5^ Hz to 10^−1^ Hz. The Nyquist plots obtained for 304L stainless steel immersed in physiological serum with and without different concentrations of HKER at 25 °C and 45 °C, respectively, are shown in Figure 2a,b. The capacitive loops are easily deviated from a semicircular shape due to surface defects and/or heterogeneities [20,32,33,34] caused by the randomly agglomeration of chemical species at the metal/electrolyte interface. Moreover, for the same studied system, more extensive capacitive loops were recorded at 25 °C (Figure 2a) compared to those at 45 °C (Figure 2b). It can be observed (Figure 2a,b) that the diameters gradually increase with the inhibitor concentration, and, consequently, larger capacitive loops are highlighted due to the adsorption of an increasing number of HKER molecules on the alloy surface. Therefore, a protective layer that covers more and more extensive zones on the surface is formed, acting as a barrier at the metal/electrolyte interface, delaying corrosion by restricting the transfer of chemical species from the electrode to the electrolyte and vice versa, thus inducing an increase in charge transfer resistance (*R*_ct_). On the other hand, the *R*_ct_ decreases with the increase in temperature caused by, generally, an increase in the number of active sites (unprotected sample) or the desorption of peptides, leading to the appearance of additional free places on which the corrosion processes can be intensified. Consequently, at the same inhibitor concentration, R_ct_ reaches higher levels at a low temperature, and, at both 25 °C and 45 °C, R_ct_ increases with an increase in the HKER concentration, in agreement with the literature data [32,35].

The Nyquist diagram (Figure 2a,b) was evaluated by fitting the experimental data using the Randles equivalent circuit (inserted in Figure 2a), consisting of the charge transfer resistance (*R*_ct_) connected in parallel with the electrical double layer capacitance (*C_dl_*), both of which are linked in series with the solution resistance (*R*_s_).

The intersection of the capacitive loop with the real axis at very low frequencies represents (*R*_ct_ + *R*_s_), and at high frequencies, it corresponds to the electrolyte resistance (*R*_s_) [36,37,38,39].

The charge transfer resistance (*R*_ct_) was used to calculate the HKER inhibition efficiency, (IE), according to Equation (1) [6,32,40].

(1) $$IE=\frac{R_{ct}-R_{ct}^{0}}{R_{ct}}\times 100$$

where $R_{ct}^{0}$ represents the charge-transfer resistance obtained after the corrosion of 304L stainless steel in a physiological serum blank solution; *R*_ct_ is the charge-transfer resistance of 304L of stainless steel corroded in physiological serum containing various HKER concentrations.

The inhibition efficiency (IE) and electrochemical parameters calculated from EIS are presented in Table 1.

The following conclusions can be deduced from Table 1: (i) *R*_ct_ increases considerably with the increase in the HKER concentration, both at 25 °C and at 45 °C; (ii) the inhibition efficiency (IE) at 25 °C is higher than that obtained at 45 °C, probably due to the occurence of a more even and coherently organized upper layer, ensuring an effective inactivation of the active surface sites at a lower temperature; (iii) the decrease in *R*_s_ and *C_dl_*, can be attributed to the ability of HKER molecules to replace pre-adsorbed molecules of water and/or other ions [32,35], such as chloride anions. 

A similar equivalent circuit was used for 304L stainless steel corroded in hydrochloric acid solution, both in the absence and presence of a composite inhibitor based on polyvinyl alcohol and silver nanoparticles [6], when the inhibition efficiency reached the value of 81.7%. In addition, the anticorrosive performance of a vinyl butyral-co-vinyl alcohol-co-vinyl acetate-based copolymer (PVBA) on stainless steel corrosion was investigated in a sodium chloride solution, resulting in a moderate inhibition efficiency of 72 ± 2% and a polarization resistance of 4.52 kΩ cm^2^ for SS in an uninhibited solution and of 15.1 kΩ cm^2^ in the presence of PVBA. This was determined by fitting the experimental data in a similar equivalent circuit [8].

Figure 3 shows the Bode impedance diagrams (Figure 3a,b) and Bode phase diagrams (Figure 3a’,b’) at 25 °C (Figure 3a,a’) and 45 °C (Figure 3b,b’).

Analyzing the Bode impedance diagrams (Figure 3a,b), it can be seen that the impedance response at the frequency of 10^−1^ Hz (log Freq = −1) follows the same trend as in the Niquist diagram. Thus, for the PS blank solution, log*Z* has the lowest values: 3.63 at 25 °C and 3.47 at 45 °C, respectively, which gradually increase to 4.53 (25 °C) and 4.21 (45 °C) for an HKER concentration of 40 g L^−1^. 

Thus, for the impedance (*Z*), almost similar values to (*R*_ct_ + *R*_s_) were identified, which confirms the validity of the equivalent circuit used to fit the experimental data [32].

From the Bode phase diagrams (Figure 3a’,b’), it can be seen that, in the presence of the HKER inhibitor, at 25 °C (Figure 3a’), an extensive phase angle maximum, centered around −79 degrees, was recorded, reaching very close values to that obtained for the physiological serum blank solution.

At 45 °C (Figure 3b’), the HKER presence leads to the phase angle maximum shifting to lower values, from −72 (PS) to around −80 (PS/HKER), more or less hypothetically attributed to the change in the surface film configuration, which can be disturbed by the temperature increase.

### 3.3. Potentiodynamic Polarization

**Figure 4:**
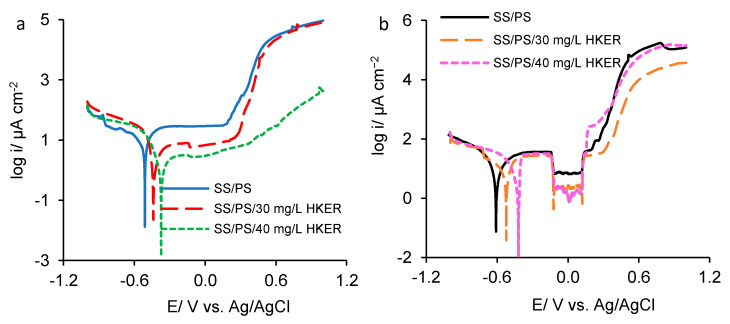
Semi-logarithmic curves recorded in a potential range between −1.0 V and 1.0 V for 304L of stainless steel corroded in physiological serum, both in the presence and absence of HKER: (**a**) at 25 °C; (**b**) at 45 °C.

The potentiodynamic polarization was performed on 304L stainless steel electrode in PS both with and without two HKER concentrations at 25 °C and 45 °C, respectively, in the potential range of −1.0 and 1.0 V. The semi-logarithmic curves were recorded and displayed in Figure 4. Both at 25 °C (Figure 4a) and at 45 °C (Figure 4b), in the presence of HKER, the polarization curves shifted in the positive direction, indicating lower current density areas, and, consequently, the corrosion potential (*E*_corr_) moved to higher values compared to that recorded in the PS blank solution.

As shown in Figure 4a, at 25 °C, a characteristic plateau, located at a lower current density, is observed in the presence of 30 mg L^−1^ HKER, compared to that recorded in the PS blank solution. This is due to the development of a protective surface layer by inhibitor adsorption, providing the substrate with a higher stability compared to that of the layer formed in the PS blank solution. Over 0.3 V, the current density sharply increases, indicating a SS trans-passivation process caused by HKER desorption from its surface.

At a concentration of 40 mg L^−1^ HKER, beyond 0.3 V, the current density exhibits a slower increase compared to the previous one. This is due to the fact that more active sites on the steel surface are occupied by HKER-adsorbed molecules, and fewer free micro-zones are formed and, therefore, corrosion processes take place less intensively. At 45 °C (Figure 4b), at a potential of −0.1 V, there is an alteration of the passive plateau, when the current density suddenly drops, which can be associated with the damage and relocation of the passive surface layer, up to 0.12 V. Beyond 0.12 V, the steel is reactivated by entering the trans-passivation zone. Consequently, in the anodic field, the polarization curves present three distinctive zones: (a) the active area, where the current densities increase directly proportional to the potentials, being accompanied by iron oxidation; (b) the passive zone, where the current density remains approximately constant, and the potential increases; (c) the trans-passive zone, where the steel is reactivated, the anodic dissolution of the iron being followed by the oxidation of nickel and chromium, which are part of the alloy. At both temperatures, the cathodic process is influenced to a lesser extent by HKER presence, indicating that it acts as a mixed inhibitor, predominantly anodic.

**Figure 5:**
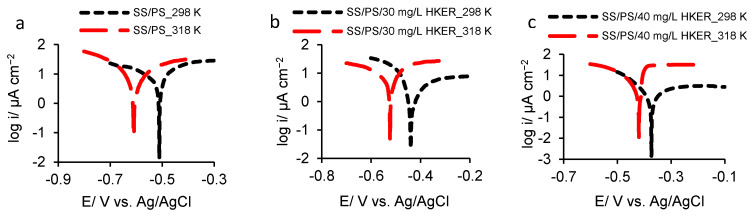
Semi−logarithmic curves recorded for 304L of stainless steel activity field to calculate corrosion current density in physiological serum, in the presence and absence of HKER, at 25 °C and 45 °C, respectively: (**a**) in physiological serum in the absence of HKER; (**b**) in physiological serum containing 30 mg L^−1^ HKER; (**c**) in physiological serum containing 40 mg L^−1^ HKER.

In order to elucidate the HKER effect on 304L stainless steel corrosion, taking into consideration its susceptibility zone in physiological serum, the semi-logarithmic polarization curves recorded at both 25 °C and 45 °C, respectively, are comparatively presented for the alloy activity zone, where iron preferentially oxidizes (Figure 5).

It is mentioned that the corrosion current density (*i*_corr_) is difficult to determine in the passive or trans-passive zones. For this reason, the change in electrochemical parameters is identified in the active zone for the Tafel segments located at potentials higher than 52 mV than the corrosion potential (*E*_corr_) for the anodic field and lower than −52 mV for the cathodic one, respectively.

The main similarities between the polarization curves consist of the following: (i) both in the absence and in the presence of the inhibitor, the corrosion potentials (*E*_corr_) recorded at 45 °C shift in the negative direction compared to those at 25 °C, and the polarization curves are located in higher current areas; (ii) the corrosion current density (*i*_corr_) was determined at the intersection of the Tafel segments extrapolated to the corrosion potential, reaching lower values at 25 °C than at 45 °C.

Certain details for the corrosion current density calculation will be provided below.

Equation (2) shows that the corrosion process rate is described by the two terms: the anodic and cathodic ones. This allows for the graphical determination of the main corrosion parameter, i_corr_, by plotting semilogarithmic polarization curves both for the anodic and for the cathodic processes, as shown in Figure 5. In the potential range, where a single partial reaction (anodic or cathodic) predominates, the polarization curve is linear (Tafel line) for pure electron transfer reactions.

(2) $$i=i_{corr}[\exp\left(\frac{zF\alpha \eta }{RT}\right)-\exp\left(-\frac{zF\beta \eta }{RT}\right)]$$

where *i*—corrosion current density; *η*—overvoltage; *z*—number of exchangeable electrons in system; α and β kinetic transfer coefficients; *α* + *β* = 1; F—Faraday’s constant = 96,500 C; *R* is the universal constant of gases = 8.31 J mol^−1^ K^−1^; *T*—temperature (K).

According to electrochemical kinetics, to determine the anodic Tafel equation, the conditions *η* ≥ 52 mV and *E* >> *E_corr_* are imposed, when the cathodic term $\exp\left(-\frac{zF\beta \eta }{RT}\right)\to 0$ and Equation (2) become as follows (3):

(3) $$i=i_{corr}[\exp\left(\frac{zF\alpha \eta }{RT}\right)]$$

For the cathodic domain, when *η* ≤ −52 mV and *E* << *E_corr_*, the anodic term $[\exp\left(\frac{zF\alpha \eta }{RT}\right)]\to 0$. Thus, Equation (2) can be written according to Expression (4).

(4) $$i=i_{corr}[\exp\left(-\frac{zF\beta \eta }{RT}\right)]$$

Successively, by logarithmization, applying a correction factor of 2.303, and rearranging the terms, the Tafel equations for both the anodic and cathodic processes are obtained, as the Expressions (5) and (6) show.

(5) $$\eta =b_{a}logi-b_{a}\log i_{corr}$$

(6) $$\eta =b_{c}logi_{corr}-b_{c}\log i$$

By solving the system of Equations (5) and (6), the coordinates of the intersection points of the Tafel lines are obtained, from which *E_corr_* and log*i_corr_* are computed. The anodic (*b_a_*) and cathodic (*b_c_*) Tafel slopes are calculated according to Equations (7) and (8).

(7) $$b_{a}=({\frac{d\eta }{dlogi})}_{\eta \gg 52mV}=\frac{2.303RT}{zF\alpha }$$

(8) $$b_{c}=(-{\frac{d\eta }{dlogi})}_{\eta \leq -52mV}=-\frac{2.303RT}{zF\beta }$$

Additionally, the potentio-gravimetric method was simultaneously applied, by converting the corrosion current density (*i_corr_*) to the corrosion rate (*CR*), according to Equation (9) [7,32].

(9) $$CR(\mu m{Year}^{-1})=\frac{i_{corr}A}{1000zF\rho }\times 24\times 365\times 10^{6}$$

where *z* is the number of electrons interchangeable in the process; *i_corr_* is the corrosion current density (A m^−2^); A represents iron atomic mass (g mol^−1^); *F* is Faraday’s constant (A h); *ρ* is iron density (kg m^−3^); 24 and 365 are multiplication factors for hours and days, respectively; 1000 and 10^6^ and are the correction factors for the density unit, from kg m^−3^ to g m^−3^, and the transformation factor for the *CR* unit from meters (m) to μm, respectively.

The gravimetric corrosion index, (*k_g_* g m^−2^ h^−1^) will be determined according to the *CR* values by applying Equation (10) [41].

(10) $$k_{g}(gm^{-2}h^{-1})=\frac{i_{corr}\times A}{zF}$$

The polarization resistance (*R_p_*) was determined using the Stern–Geary Equation (11) [42]:

(11) $$R_{p}(\Omega {cm}^{2})=\frac{b_{a}\times b_{c}}{2.303(b_{a}+b_{c})}\times \frac{1}{i_{corr}}$$

where *i_corr_* is the corrosion current density (A cm^−2^) and *b_a_* and *b_c_* are the anodic and cathodic Tafel slopes (V dec^−1^).

The electrochemical parameters, namely the corrosion potential (*E_corr_*) and corrosion current density (*i_corr_*), as well as their conversion to corrosion rate (*CR*) and polarization resistance (*R_p_*), were computed using VoltaMaster 4 software. The electrochemical parameters, k_g_, *CR* and *IE* are listed in Table 2.

The inhibition efficiency (*IE*/%) of HKER on the 304L stainless steel corrosion in physiological serum was determined according to the values of i_corr_ [7,43,44], CR [7,45,46] and R_p_ [1,7,17,43,44], before and after potentiodynamic polarization, using Equations (12)–(14).

(12) $$IE=\frac{i_{corr}^{o}-i_{corr}}{i_{corr}^{o}}\times 100$$

(13) $$IE=\frac{{CR}^{o}-CR}{{CR}^{o}}\times 100$$

(14) $$IE=\frac{R_{p}-R_{p}^{o}}{R_{p}}\times 100$$

The average inhibition efficiency (*IE_m_*) was calculated as the arithmetic mean of the inhibition efficiencies obtained according to Equations (12)–(14).

Analyzing the potentiodynamic polarization data displayed in Table 2, the following conclusions are noted: (i) at both temperatures, *i_corr_*, *k_g_* and *CR* decreased, while *R_p_* and *IE* increased with an increasing HKER concentration; (ii) inhibition efficiency reached the highest level (*IE_m_* = 88.5%) at 25 °C and 40 mg L^−1^ HKER; (iii) at 45 °C, for the same inhibitor concentration, *i_corr_*, *k_g_* and *CR* increases, and *R_p_* and *IE_m_* decreases compared to the values obtained at 25 °C, probably due to the fact that partial HKER desorption is relatively favored by a temperature increase; at 45 °C, *IE_m_* reaches the value of 80.6%, approximately 8% lower than that calculated at 25 °C; (iv) the results are in full agreement with those obtained from the EIS.

### 3.4. Activation Energy Calculations: Determination of Thermodynamic Activation Parameters

To calculate the apparent activation energy (*E*_a_), taking into account *k_g_* (g m^−2^ h^−1^), the linearized form of the Arrhenius Equation (15) [42,47,48,49,50] is applied for the two studied temperatures, Equations (15)–(18).

(15) $$lnk_{g}=lnA-\frac{E_{a}}{R}\cdot \frac{1}{T}$$

where *A* is the Arrhenius pre-exponential factor, *T* is the absolute temperature (K) and *R* is the universal gas constant (8.31 J mol^−1^ K^−1^).

(16) $$lnk_{g1}=lnA-\frac{E_{a}}{R}\cdot \frac{1}{T_{1}}$$

(17) $$lnk_{g2}=lnA-\frac{E_{a}}{R}\cdot \frac{1}{T_{2}}$$

where *T_1_* = 25 °C and *T_2_* = 45 °C; *k_g1_* and *k_g2_* represent the gravimetric corrosion indices at 25 °C and 45 °C, respectively.

By subtracting Equations (16) and (17) and after rearranging the terms, the activation energy expression (*E_a_*) is obtained (18).

(18) $$E_{a}=\frac{R\cdot T_{1}\cdot T_{2}\cdot ln\frac{k_{g1}}{k_{g2}}}{T_{1}-T_{2}}$$

The activation energy varies as follows:

*E_a_*_(PS)_ = 26.62 kJ mol^−1^ < *E_a_*_(PS/30 mg L_^−1^_HKER)_ = 43.25 kJ mol^−1^ < *E_a_*_(PS/40 mg L_^−1^_HKER)_ 47.4 kJ mol^−1^.

The activation energy, *E_a_*, of stainless steel immersed in a PS blank solution has a lower value than that determined in PS containing HKER, suggesting a good adsorption of the inhibitor [47] on the alloy surface. However, it does not reach the required threshold of 80 kJ mol^−1^, which involves chemical adsorption [47]. It has also been reported that a higher *E_a_* value in the presence of an inhibitor, compared to that of the blank solution, usually indicates physisorption [42,48]. Consequently, HKER acts through a physical adsorption mechanism or through a mixed mechanism, where physisorption prevails over chemisorption.

To confirm the activation energy evolution, the corrosion rate was determined using the potentio-gravimetric method under similar conditions to those previously applied, for various HKER concentrations, such as 10 mg L^−1^; 15 mg L^−1^; 20 mg L^−1^; 25 mg L^−1^; 30 mg L^−1^; 35 mg L^−1^; 40 mg L^−1^, and at four temperatures, namely, 25 °C, 35 °C, 45 °C and 55 °C. The data are centralized in Table 3. As can be seen, the corrosion rate decreases with an increasing inhibitor concentration and a decrease of temperature.

Thus, to determine the activation energy from Equation (8), ln*k_g_* = f (1/*T*) was plotted, obtaining straight lines with slopes of −*E_a_*/*R*, and the intersection with the *y*-axis representing ln*A*, the linearity coefficient R^2^ being close to unity.

**Figure 6:**
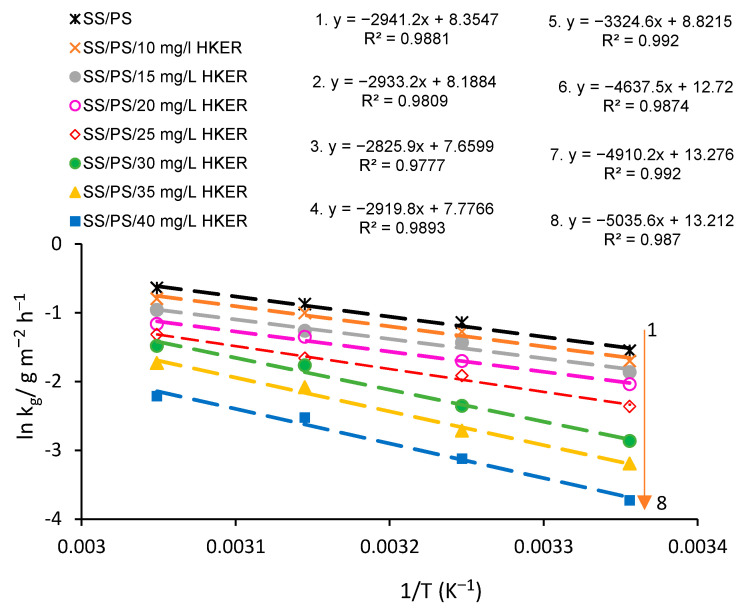
Arrhenius diagram obtained for 304L stainless steel corroded in physiological serum both in the absence and presence of different HKER concentrations.

Figure 6 shows the Arrhenius diagram and the equations related to the straight lines drawn for the HKER studied concentrations. By deriving the equations inserted in Figure 6, the slope (dy/dx) equal to −*Ea*/*R* is obtained, from which *E_a_*, listed in Table 4, is determined from Equation (19).

(19) $$E_{a}=-R(\frac{dy}{dx})$$

Inspecting the data from Table 4, it can be seen that E_a_ gradually increases with an increased inhibitor concentration, starting with 25 mg L^−1^ HKER in physiological serum. The HKER concentration’s impact on *E_a_* values indicates that a complex compound has been formed between the inhibitor molecules and cations from the stainless steel surface [48]. At concentrations lower than 25 mg L^−1^ HKER, the activation energy reached values almost similar to those obtained for stainless steel corroded in a physiological serum blank solution. Thus, the same energy barrier intervenes in the corrosion process, suggesting that an insufficient HKER concentration leads to the occurrence of numerous active surface sites on which the corrosion process similarly continues, as in the physiological serum blank solution. 

On the other hand, the Arrhenius pre-exponential factor (*A*) progressively decreases as the HKER concentration increases, reaching the lowest value for the SS/PS/20 mg L^−1^ HKER system. At HKER concentrations higher than 20 mg L^−1^, the Arrhenius factor (*A*) significantly rises, suggesting an enhancement in the frequency of collisions between the inhibitor molecules and the alloy surface and, therefore, an increased number of adsorbed water molecules are replaced by HKER molecules.

Moreover, for an *E_a_* graphically calculated (Figure 6), values close to those determined from Equation (18) were obtained, for inhibitor concentrations of 30 mg L^−1^ and 40 mg L^−1^, showing the Arrhenius model’s validity applied on corrosion inhibition of 304L stainless steel in PS, both in the absence and presence of the HKER inhibitor.

As an alternative to the Arrhenius model, the transition-state equation will be applied for HKER concentrations varying from 25 mg L^−1^ to 40 mg L^−1^. The linearized form of the transition-state equation is given by Expression (20) [42,47,48,49]:

(20) $$ln\frac{k_{g}}{T}=ln\frac{R}{N\cdot h}+\frac{\Delta S_{a}}{R}-\frac{\Delta H_{a}}{R}\cdot \frac{1}{T}$$

where Δ*H_a_* (kJ mol^−1^) is the enthalpy of activation and Δ*S_a_* (J mol^−1^ K^−1^) is the entropy of activation, respectively; *k_g_* is the corrosion rate, expressed as the gravimetric corrosion index, *h* is Planck’s constant, 6.626 × 10^−34^ J s; *N* is Avogadro’s number, 6.023 × 10^23^ mol^−1^; *T* is the absolute temperature (K); *R* is the universal gas constant (8.31 J mol^−1^ K^−1^).

**Figure 7:**
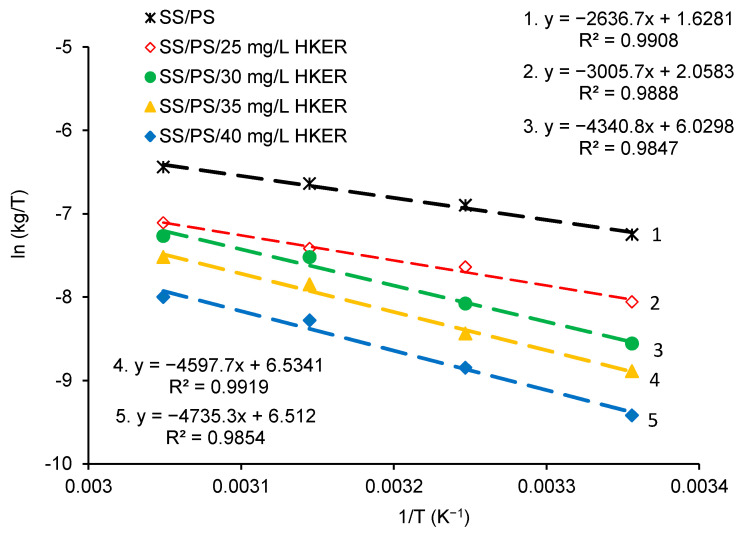
Transition state diagram obtained for 304L stainless steel corroded in physiological serum both in the absence and presence of different HKER concentrations.

Figure 7 shows the plot of ln(*k_g_*/*T*) as a function of 1/*T*, when straight lines were obtained, with the slopes representing −Δ*H_a_/R*. From the ln(*k_g_/T*)–axis intercepts, [ln(*R/h·N*) + Δ*S_a_/R*] values were calculated and are given in Table 4. Consequently, using the Equations inserted in Figure 7, Δ*H_a_* and Δ*S_a_* were computed, with relations (21) and (22).

(21) $${\Delta H}_{a}=-R(\frac{dy}{dx})$$

(22) $$\Delta S_{a}=R[y\left(x=0\right)-ln\left(\frac{R}{N\cdot h}\right)]$$

The positive values of Δ*H_a_* gradually increase with an increased HKER concentration, from 21.91 kJ mol^−1^ (SS/PS) to 39.95 (SS/PS/40 mg L^−1^ HKER), revealing an endothermic dissolution process of stainless steel [42,49]. The decline in corrosion rate of 304L stainless steel is mainly controlled by the activation thermodynamic parameters [47].

The negative activation entropy (Δ*S_a_*) values indicate that, in the determining stage of the reaction rate, the activated complex constitutes more of an association step than a dissociation one, meaning that, on passing from reactants to the activated complex, a decrease in disorderliness takes place [42,47,48].

The increase of Δ*S_a_* from −183.91 J mol^−1^ K^−1^ to −143.33 J mol^−1^ K^−1^ (from higher to lower negative values), observed with an increased HKER concentration, shows an increase in disorder occurring during HKER adsorption, which is probably due to the replacement of other adsorbed chemical species (water, Cl^−^) on the stainless steel surface by the inhibitor molecules [47].

### 3.5. Adsorption Isotherm Approach. Calculation of Adsorption Parameters

First, the degree of 304L stainless steel surface coverage (*θ* = IE/100) was determined using Equation (23). For concentrations lower than 25 mg L^−1^ HKER, the corrosion rate decreased with an increase in inhibitor concentration and a decrease in temperature, but the surface coverage degree varied randomly (Table 5) due to an insufficient inhibitor concentration, leading to the formation of a discontinuous and dispersed protective film on the metal surface. At concentrations higher than 25 mg L^−1^, θ increased consistently with the inhibitor concentration and decreased with an increase in temperature for the same concentration of HKER.

(23) $$\theta =\frac{{CR}^{o}-CR}{{CR}^{o}}$$

The most well-known way to quantitatively reproduce an inhibitor’s adsorption on the metal surface is represented by fitting the experimental data with a certain suitable adsorption model, obtaining regression coefficients (R^2^) very close to unity. Therefore, Langmuir and Freundlich adsorption isotherms were firstly applied for the HKER adsorption on the 304L stainless steel surface, using their linearized forms represented by Equations (24) and (25), respectively [32,48].

(24) $$\frac{C}{\theta }=C+\frac{1}{K_{ads}}$$

(25) $$log\theta =nlogC+logK_{ads}$$

*θ* is the surface coverage degree; *K_ads_* is the adsorption–desorption equilibrium constant; *C* represents the inhibitor concentration; n represents a Freundlich factor.

**Figure 8:**
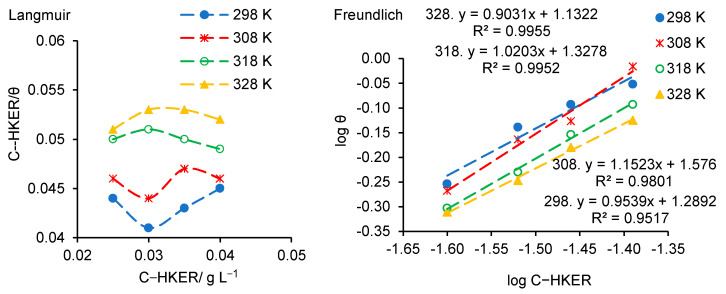
Langmuir and Freundlich diagrams obtained for HKER adsorption on 304L stainless steel surface physiological serum, at different temperatures.

The Langmuir and Freundlich plots are displayed in Figure 8. It can be seen that the Langmuir isotherm (Figure 8, Langmuir) is unsuitable for the linearization of the experimental data and implicitly for HKER adsorption on the stainless steel surface. The Freundlich isotherm (Figure 8, Freundlich) does not accurately represent an adsorption model for HKER at temperatures of 298 K and 308 K, respectively, the regression coefficients (R^2^) being lower than 0.99.

In the following, the Temkin isotherm and El-Awady’s adsorption model will be comparatively presented using their respective linear expressions, represented by Equation (26) (Temkin) [32,51,52] and Equation (27) (El-Awady) [32,53], respectively.

(26) $$\theta =\frac{1}{f}lnC+\frac{1}{f}lnK_{ads}$$

(27) $$log\frac{\theta }{1-\theta }=ylogC+logK$$

*K_ads_* = *K*^1/y^ (28)

where *f* represents a positive factor that characterizes the surface heterogeneity [32,50,51,54]; *y* is the number of water molecules replaced by one inhibitor molecule [53]; K_ads_ is the adsorption–desorption equilibrium constant; *C* represents the inhibitor concentration.

**Figure 9:**
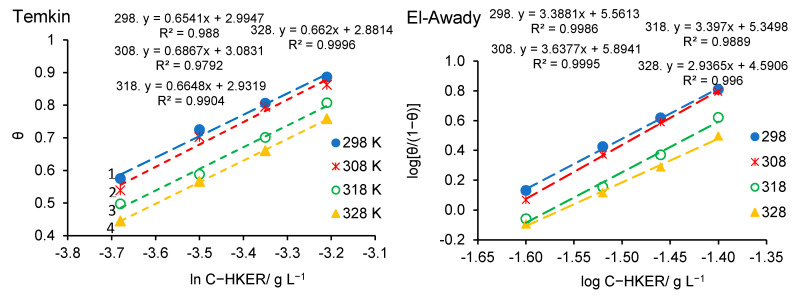
Temkin and El-Awady’s models obtained for HKER adsorption on 304L stainless steel surface in physiological serum, at different temperatures.

As can be seen from Equation (26), by plotting *θ* = *f* [ln(*C*−HKER)], straight lines are obtained, with the slope equal to 1/*f* and the intercept equal to [(1/*f*)ln*K_ads_*], from which *K_ads_* is easily deduced [32,51,52]. By analogy, using Equation (27), from the plot of $log\frac{\theta }{1-\theta }$ = *f*(log*C*), straight lines are obtained, with *y*-slopes and intercept equal to log*K*. *K_ads_* is computed according to Equation (28) [53]. Both models are presented in Figure 9.

As shown in Figure 9, the experimental data obey both adsorption models, except at the temperature of 308 K, for which the coefficient R^2^ reaches a lower value of 0.9792. Therefore, the El-Awady model provides a slightly improved fitting of the experimental data, constituting a priority option for HKER adsorption on the steel surface, in relation to the Temkin isotherm.

The standard Gibbs free energy of adsorption ($\Delta G_{ads}^{o}$) was determined using Equation (29) [32,42,48,55].

(29) $$\Delta G_{ads}^{o}=-RTln(55.5\cdot K_{ads})$$

where *R* is the universal constant of gases (8.31 J mol^−1^ K^−1^), *T* is the temperature and 55.5 is the value of the molar concentration of water in the solution.

In this study, the adsorption–desorption equilibrium constant (*K_ads_*) is expressed in L g^−1^. Thus, the concentration of water in the solution must be considered in g L*^−^*^1^, meaning 55.5 mol × 18.015283 g mol*^−^*^1^ ≈ 1000 g L*^−^*^1^ [56]. Equation (29) can be written as Equation (30) [56].

(30) $$\Delta G_{ads}^{o}=-RTln(1000\cdot K_{ads})$$

The values of $\Delta G_{ads}^{o}$ and R^2^ are listed in Table 6. It is observed that relatively close values were obtained for $\Delta G_{ads}^{o}$, revealing a spontaneous adsorption process, and the physical adsorption prevails over the chemical one.

The adsorption parameters, enthalpy ($\Delta H_{ads}^{o}$, kJ mol^−1^) and entropy ($\Delta S_{ads}^{o}$, J mol^−1^ K^−1^), were be calculated using Equations (31) and (32) [42]:

(31) $$\Delta G_{ads}^{o}=\Delta H_{ads}^{o}-T\Delta S_{ads}^{o}$$

(32) $$lnK_{ads}=-\frac{\Delta H_{ads}^{o}}{R}\cdot \frac{1}{T}+\frac{\Delta S_{ads}^{o}}{R}-ln1000$$

**Figure 10:**
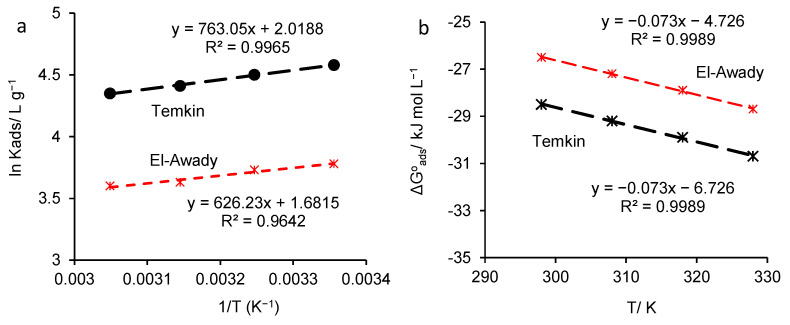
The determination of adsorption parameters (${∆H}_{ads}^{o}$ and ${∆S}_{ads}^{o}$) for 304L stainless steel corroded in physiological serum containing various HKER concentrations, (**a**) the plot of ln*K_ads_* = f(1/T), (**b**) ${∆G}_{ads}^{0}$ linear variation over T.

Figure 10a displays the plot of ln*K_ads_* = f(1/*T*) for HKER adsorption on the stainless steel surface. From the slope of the straight line ($-\Delta H_{ads}^{o}$**/***R*), adsorption enthalpy (${∆H}_{ads}^{0}$) was calculated, and from the intercept [($\Delta S_{ads}^{o}$/*R*) − ln(1000)], adsorption entropy was determined. Figure 10b represents the ${∆G}_{ads}^{0}$ linear variation over temperature, where the slope is associated with $-\Delta S_{ads}^{o}$ (kJ mol^−1^ K^−1^) and the intercept with $\Delta H_{ads}^{o}$ (kJ mol^−1^). As Table 6 shows, by applying the two methods of calculation, very close values were obtained for the enthalpy and entropy of adsorption, confirming the validity of the proposed adsorption model. Furthermore, from Figure 10a, it can be observed that the adsorption parameters ($\Delta H_{ads}^{o}$ and $\Delta S_{ads}^{o}$) were obtained with a lower linearity coefficient (R^2^ = 0.9642) when *K_ads_* was calculated from El-Awady’s model, compared to 0.9965 when *K_ads_* was determined from the Temkin isotherm. However, the plot of ${∆G}_{ads}^{0}$ = f(T) for both models shows a high R^2^ value (0.9989). For the determination of *K_ads_*, the El-Awady model offers a higher degree of confidence, with R^2^ ranging between 0.9889 and 0.9995, compared to the Temkin isotherm when R^2^ falls below 0.98. Accordingly, further comments will refer to the results obtained from the El-Awady’s adsorption model.

As shown in Table 6, the negative and closely related values for $\Delta G_{ads}^{o}$ were obtained, slightly decreasing from −26.5 kJ mol L^−1^ at 298 K to −28.7 kJ mol L^−1^ at 328 K, which indicates the stability of the protective layer [47,48] and the spontaneous adsorption of HKER [42,47,48] on the stainless steel surface, involving an endothermic process [47]. The values of ${∆G}_{ads}^{0}$ did not reach the threshold of −40 kJ mol^−1^ imposed for chemical adsorption and, also, the degree of surface coverage (θ) and the inhibition efficiency (IE), respectively, decreased with an increase in the temperature, indicating that HKER acts through a predominantly physical adsorption mechanism [47].

The negative value of $\Delta H_{ads}^{o}$ can suggest either physisorption, chemisorption or a mixture of both types of adsorption [42]. The positive value of $\Delta S_{ads}^{o}$ can result from the replacement process of water molecules adsorbed on the metal surface with inhibitor molecules, leading to an increase in the disorder of the adsorption process [42].

Another study reported that for three newly synthesized dipeptide Schiff bases formed by the condensation glycyl-l-tyrosine and indole-3-carboxaldehyde (GTI), the same aldehyde and glycyl-glycine (GGI), or glycyl-l-glutamine (GGMI), relatively close values of ${\Delta G}_{ads}^{o}$ were calculated, being investigated as corrosion inhibitors for mild steel in a 1.0 mol L*^−^*^1^ HCl solution. Thus, for GGI, ${\Delta G}_{ads}^{o}$ slightly decreased from −33.26 kJ mol*^−^*^1^ to −36.56 kJ mol*^−^*^1^ in the temperature range between 293 K and 323 K, whereas for the other two, in the same temperature range, similar slight variations of the ${\Delta G}_{ads}^{o}$ values were obtained, from −33.78 kJ mol*^−^*^1^ to −36.28 kJ mol*^−^*^1^ (GGMI) and from to −34.73 kJ mol*^−^*^1^ to −36.6 kJ mol*^−^*^1^ (GTI), respectively. Similar to HKER, the ${\Delta G}_{ads}^{o}$ value slightly declined with increasing temperature [57]. The differences between the values are caused by substrate type and the environment composition. A natural polymer (okra pectin) has been reported as a corrosion inhibitor for 304 stainless steel (304 SS) in a 1.0 mol L*^−^*^1^ HCl solution [56]. The ${\Delta G}_{ads}^{o}$ values between −23 kJ mol*^−^*^1^ and −24 kJ mol*^−^*^1^ were obtained, in the temperature range from 25 to 50 °C [56]. Additionally, for the corrosion inhibition of low-carbon steel using albumin, ${\Delta G}_{ads}^{o}$ reached a value lower than −20 kJ mol*^−^*^1^, demonstrating the spontaneity of the adsorption process and the predominant mechanism of physisorption [58]. Consequently, our study falls within the limits reported by certain authors for adsorption parameters and the HKER action mechanism.

### 3.6. Surface Characterization

#### 3.6.1. Optical Microscopy

**Figure 11:**
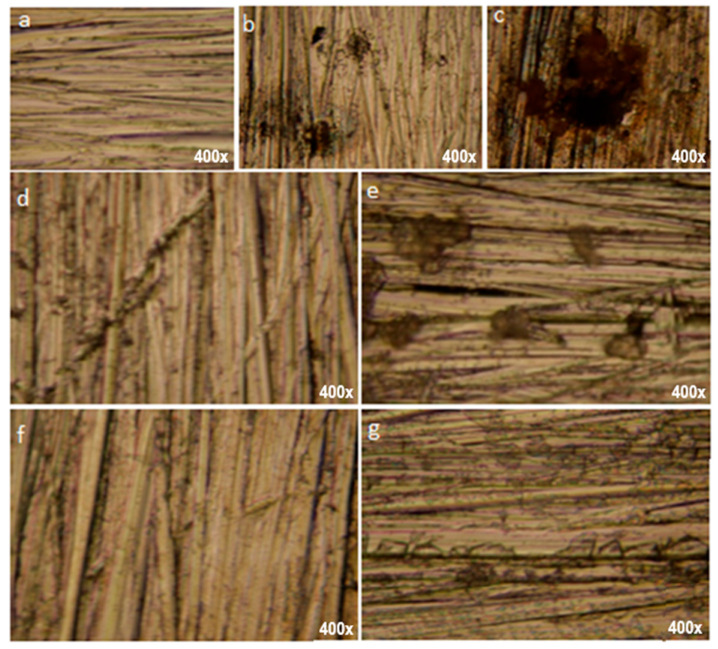
Optical microscopy images acquired for 304L stainless steel surface: (**a**) before corrosion (control sample); (**b**) after corrosion in PS blank solution, at 25 °C; (**c**) after corrosion in PS blank solution, at 45 °C; (**d**) after corrosion in PS containing 30 mg L^−1^ HKER, at 25 °C; (**e**) after corrosion, in PS containing 30 mg L^−1^ HKER, at 45 °C; (**f**) after corrosion in PS containing 40 mg L^−1^ HKER, at 25 °C; (**g**) after corrosion in PS containing 40 mg L^−1^ HKER, at 45 °C.

The microscopic images of 304L stainless steel, before and after potentiodynamic polarization, carried out in PS both with and without two concentrations of HKER, are displayed in Figure 11. The control sample surface shows a characteristic morphology of the stainless steel surface before potentiodynamic polarization (Figure 11a). The microscopic images from Figure 11b (SS/PS, 25 °C) and Figure 11c (SS/PS, 45 °C) show that, after the electrochemical measurements, the alloy surface was coated with large corrosion spots, which changed its appearance, this change being more nuanced at 45 °C (Figure 11c) than at 25 °C (Figure 11b).

Moreover, some fractions of the surface retain the morphology of the standard, indicating that the passive layer formed during electrochemical measurements (Figure 3) is not completely damaged during the trans-passivation range (Figure 3), providing a protective effect to the stainless steel surface, especially at 25 °C (Figure 11b).

In Figure 11d–g, the microscopic images are completely different compared to those presented above, showing less pronounced disturbances of the surface layer morphology.

Figure 11e (SS/PS/30 mg L^−1^ HKER, 45 °C) and Figure 11g (SS/PS/40 mg L^−1^ HKER, 45 °C) display a more damaged appearance of the surface than the corresponding ones at 25 °C (Figure 11d,f), highlighting that the increase in temperature causes a slightly desorption of the inhibitor.

On the other hand, a more coherent organized surface layer can be observed at 25 °C in the case of the SS/PS/40 mg L^−1^ HKER system (Figure 11d) compared to the one corresponding to SS/PS/30 mg L^−1^ (Figure 11f).

#### 3.6.2. Atomic Force Microscopy (AFM)

**Figure 12:**
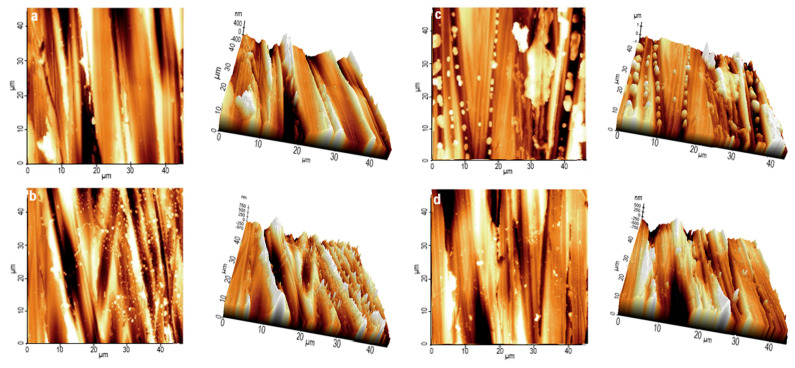
Two−dimensional and three−dimensional AFM images acquired for 304L stainless steel surface at 25 °C: (**a**) before corrosion (control sample); (**b**) after corrosion in PS blank solution; (**c**) after corrosion in PS containing 30 mg L^−1^ HKER; (**d**) after corrosion in PS containing 40 mg L^−1^ HKER.

The 2D and 3D images of 304L stainless steel morphologies after the potentiodynamic polarization in PS at 25 °C are depicted in Figure 12. In the absence of HKER (Figure 12b), the surface displays an irregular topography caused by the PS corrosion attack. Some microdeposits can be distinguished that affect the appearance, uniformity and smoothness of surface, compared to control sample (Figure 12a).

In the presence of 40 mg L^−1^ HKER in PS (Figure 12d), a smoother surface was designed at 25 °C compared to that obtained at 30 mg L^−1^ HKER concentration (Figure 12c). To confirm the previously discussed findings, the AFM roughness parameters were calculated, and the corrosion inhibition process was reported for their values. Thus, the average roughness (*R_a_*), root-mean-square roughness (*R_q_*) and maximum peak-to-valley height (*R_p−v_*) were also analyzed in other studies, indicating the corrosion inhibition [44,59,60,61]. The values of *R_a_*, *R_q_* and *R_p−v_* are listed in Table 7.

The addition of HKER in PS leads to changes in the AFM parameters of the stainless steel surface, revealing that *R_a_*, *R_q_* and *R_p−v_* reached values close to the standard but lower than those obtained for the sample corroded in physiological serum without an inhibitor. The decline in the AFM parameters indicates that a smoother surface [44,60] was obtained for the 304L stainless steel immersed in PS containing HKER, suggesting that a protective layer was formed by HKER adsorption on the alloy surface.

### 3.7. HKER Action Mechanism

The HKER action mechanism is complex due to the fact that the inhibitor consists of a multicomponent system that adsorbs onto the steel surface. Thus, the inhibitor action mechanism is the result of a synergic adsorption process, involving the constituents from a system containing a peptide molecular group and some residual amino acids, especially cysteine.

The molecular groups of peptides are involved in various donor–acceptor processes, depending on the amino acids constituting the peptide chain, which also have a significant impact on the adsorption process and corrosion inhibition, respectively. The peptide group (O=C–NH) possesses lone pair electrons at the oxygen and nitrogen atoms interacting with the vacant d-orbitals of the iron from the metallic network forming coordinative bonds and, thus, creating the premise of chemical adsorption. At the same time, cystine sulfur (–S–S–) amplifies the adsorption on the steel surface.

Furthermore, the peptide group repeats itself successively along the molecular chain, constituting additional active centers that can stimulate the interaction with the metal surface. Consequently, the adsorption process optimization takes places by blocking several active surface sites with a single peptide molecule [21]. The electron density distribution around certain groups of atoms can determine the appearance of the electrostatic interactions between the peptide chain and the metal matrix cations.

On the other hand, the organic functional groups, such as carboxyl (–COOH), hydroxyl (–OH), amine (–NH_2_) and thiol (–SH) provide electrons to the iron empty orbital, forming complexes with metal bivalent ions from the electrical double-layer. These complexes adsorb onto the surface substrate through van der Waals bonds and develop a hygroscopic protective film. Based on the experimental results, it was found that HKER acts through a mixed mechanism, and physisorption prevails over chemisorption.

Therefore, the apparent activation energy (*E_a_*) reached higher values in the presence of the inhibitor compared to those obtained in the uninhibited physiological serum, suggesting that a complex compound by HKER components–stainless steel interaction was formed [48], but the threshold required for chemical adsorption (80 kJ mol*^−^*^1^) [47] was not reached.

The activation entropy (Δ*S_a_*) increases with a rise in HKER concentration, implicitly causing the proliferation of disorder during HKER adsorption due to the replacement of other pre-adsorbed chemical species on the steel surface [47]. Additionally, negative values for ${∆G}_{ads}^{0}$ were obtained, slightly decreasing from −26.5 kJ mol L*^−^*^1^ at 298 K to −28.7 kJ mol L*^−^*^1^ at 328 K, indicating the protective layer’s stability, predominantly formed by physical adsorption [47,48].

Consequently, a mixed mechanism, wherein physisorption prevails over chemisorption, is proposed for the interaction of HKER compounds with 304L stainless steel surface.

## 4. Conclusions

In this work, electrochemical measurements, optical microscopy and AFM were performed to evaluate the inhibitory and adsorption properties of hydrolyzed keratin peptides (HKER) on stainless steel in physiological serum, revealing their action mechanism. The electrochemical measurements were made at 298 K and 308 K for HKER concentrations of 30 g L^−1^ and 40 g L^−1^. To determine the activation and adsorption parameters, the potentio-gravimetric method was applied at four temperatures, in the range of 298–328 K, the inhibitor concentration varying from 10 g L^−1^ to 40 g L^−1^ of HKER.

The electrochemical impedance spectroscopy (EIS) analysis showed that the charge transfer resistance (*R_ct_*) gradually increased with the inhibitor concentration and decreased with the rising temperature.

The potentiodynamic polarization (PDP) analysis revealed that the HKER presence in physiological serum influenced to a greater extent the metal oxidation reaction than the reduction reaction by blocking the active places on the stainless steel surface, acting as a mixed inhibitor, but mostly anodic. The corrosion current density (*i_corr_*) and corrosion rate (*CR*), respectively, progressively increased with the temperature and decreased directly proportional to the inhibitor concentration.

The potentiodynamic polarization analysis confirmed the inhibition efficiency values obtained from EIS, reaching a maximum level of around 88% at 25 °C.

The activation parameters (*E_a_*, ${∆H}_{a}$ and ${∆S}_{a}$) show that HKER acts by adsorption onto the steel surface, involving a mixed mechanism where physisorption prevails over chemisorption.

The experimental data best fit the El-Awady adsorption model, from which the adsorption–desorption constant was determined, and the standard adsorption free energy ($\Delta G_{ads}^{o}$) was implicitly calculated, indicating a spontaneous, predominantly physical, adsorption of HKER molecules on the alloy surface.

By correlating the values of the activation energy and, respectively, of the activation and adsorption thermodynamic parameters, it can be stated that the HKER mixed action mechanism takes place by physisorption, defined by van der Waals forces and to a lesser extent by chemical adsorption, which involves the formation of chemical bonds between the unshared electrons from heteroatoms and the vacant *d*-orbitals of iron.

The occurrence of a protective inhibitor coating formed by the adsorption of HKER molecules on the steel surface was supported by optical microscopy and confirmed by atomic force microscopy (AFM). The AFM parameters showed that a smoother surface was obtained for the 304L stainless steel immersed in PS containing HKER, suggesting that the protective layer was formed by HKER adsorption on the alloy surface.

The study of corrosion inhibition activity represents a good approach for investigating the impact caused by different compounds on the metal surface, allowing the design of an overview of the chemical structure profiles of perspective inhibitors.

This work highlights the inhibitory effect that hydrolyzed keratin peptides have on stainless steel corrosion, reporting some outcomes for the perspective use of corrosion inhibitors derived from proteins.

## Figures and Tables

**Table 1:** Inhibition efficiency and EIS parameters obtained from EIS for 304L stainless steel immersed in physiological serum with and without HKER at 25 °C and 45 °C, respectively.

Sample	OCP/mV vs. Ag/AgCl	25 °C						
		Nyquist Parameters			Bode Parameters			IE/ %
		*R_s_*/ Ω cm^2^	*R_ct_*/ kΩ cm^2^	*C_dl_*/ μF cm^−2^	log Z/ Ω cm^2^	Z/ kΩ cm^2^	Phase/ Degrees	
SS/PS	−209.3 ± 15.6	256.7 ± 25.3	4.1 ± 1.2	363.7 ± 19.3	3.63 ± 0.01	4.27 ± 0.1	−79.2 ± 2.3	-
SS/PS/30 mg L^−1^ HKER	−177.1 ± 12.2	234.5 ± 24.6	13.5 ± 3.6	247.2 ± 13.6	4.15 ± 0.01	14.12 ± 0.32	−79.03 ± 1.9	69.6 ± 0.9
SS/PS/40 mg L^−1^ HKER	−89.8 ± 10.8	222.9 ± 23.8	32.1 ± 6.8	213.8 ± 13.2	4.53 ± 0.01	33.8 ± 0.78	−79.64 ± 1.9	87.2 ± 0.8
**Sample**	**OCP/mV** **vs.** **Ag/AgCl**	**45 °C**						
		**Nyquist Parameters**			**Bode Parameters**			**IE/** **%**
		***R_s_*** **/** **Ω cm^2^**	***R_ct_*** **/** **kΩ cm^2^**	***C_dl_*** **/** **μF cm^−2^**	**log Z/** **Ω cm^2^**	**Z/** **kΩ cm^2^**	**Phase/** **Degrees**	
SS/PS	−232 ± 22	326.4 ± 28.1	2.8 ± 0.8	394.5 ± 37.1	3.47 ± 0.01	2.95 ± 0.07	−72.69 ± 4.1	-
SS/PS/30 mg L^−1^ HKER	−201 ± 18	296.7 ± 25.4	7.2 ± 2.0	312.8 ± 16.1	3.87 ± 0.01	7.41 ± 0.18	−80.19 ± 1.8	61.1 ± 0.4
SS/PS/40 mg L^−1^ HKER	−120 ± 13	267.2 ± 24.2	15.3 ± 3.8	292.6 ± 15.7	4.19 ± 0.01	15.48 ± 0.36	−79.76 ± 1.8	81.7 ± 0.6

**Table 2:** Inhibition efficiency, corrosion rate and electrochemical parameters obtained from the potentiodynamic polarization for 304L stainless steel corroded in physiological serum both with and without HKER, at 25 °C and 45 °C, respectively.

Sample	25 °C								
	Electrochemical Parameters			Corrosion Rate		*IE*/%			*IE_m_*/ %
	*E_corr_*/mV vs./AgCl	*i_corr_*/ μA cm^−2^	*R_p_*/ kΩ cm^2^	*k_g_*/ g m^−2^ h^−1^	*CR*/ μm Year^−1^	From Equation (12)	From Equation (13)	From Equation (14)	
SS/PS	−511 ± 15	19.95 ± 3.2	3.3 ± 0.5	0.211	237 ± 37	-	-	-	
SS/PS/30 mg L^−1^ HKER	−439 ± 15	5.37 ± 0.9	11.9 ± 2.1	0.057	65 ± 10.5	73.1 ± 0.2	72.6 ± 0.2	72.3 ± 0.6	72.7 ± 0.3
SS/PS/40 mg L^−1^ HKER	−373 ± 15	2.24 ± 0.7	27.8 ± 2.3	0.024	27 ± 8.2	88.7 ± 0.3	88.7 ± 0.5	88.1 ± 0.8	88.5 ± 0.5
**Sample**	**45 °C**								
	**Electrochemical Parameters**			**Corrosion Rate**		***IE*** **/%**			***IE_m_*** **/** **%**
	***E_corr_*** **/mV** **vs./AgCl**	***i_corr_*** **/** **μA cm^−2^**	***R_p_*** **/** **kΩ cm^2^**	***k_g_*** **/** **g m^−2^ h^−1^**	***CR*** **/** **μmY^−1^**	**From** **Equation (12)**	**From** **Equation (13)**	**From** **Equation (14)**	
SS/PS	−609 ± 28	39.8 ± 5.4	2.4 ± 0.4	0.415	462 ± 62.9	-	-	-	-
SS/PS/30 mg L^−1^ HKER	−523 ± 28	16.4 ± 2.8	5.8 ± 0.9	0.171	190 ± 32.6	58.8 ± 1.3	58.9 ± 1.4	58.6 ± 0.6	58.8 ± 1.1
SS/PS/40 mg L^−1^ HKER	−420 ± 28	8.1 ± 1.3	12.9 ± 1.8	0.08	89 ± 15.1	79.6 ± 0.5	80.7 ± 0.6	81.4 ± 0.5	80.6 ± 0.5

**Table 3:** Corrosion rate expressed in μm year^−1^ (*CR*) and in g m^−2^ h^−1^ (*k_g_*) determined by potentio- gravimetric method for 304L stainless steel corroded in physiological serum, both in the absence and presence of various HKER concentrations, at 25 °C, 35 °C, 45 °C and 55 °C.

Sample	*CR*/μm Year^−1^				*k_g_*/g m^−2^ h^−1^			
	25 °C	35 °C	45 °C	55 °C	25 °C	35 °C	45 °C	55 °C
SS/PS/	237	356	462	583	0.211	0.319	0.415	0.523
SS/PS/10 mg L^−1^ HKER	203	311	408	501	0.182	0.279	0.366	0.450
SS/PS/15 mg L^−1^ HKER	173	265	315	426	0.155	0.238	0.282	0.383
SS/PS/20 mg L^−1^ HKER	145	203	289	347	0.130	0.182	0.259	0.312
SS/PS/25 mg L^−1^ HKER	105	164	232	298	0.094	0.147	0.190	0.268
SS/PS/30 mg L^−1^ HKER	65	106	190	253	0.057	0.095	0.171	0.227
SS/PS/35 mg L^−1^ HKER	46	73	138	197	0.041	0.066	0.124	0.177
SS/PS/40 mg L^−1^ HKER	27	49	89	141	0.024	0.044	0.08	0.109

**Table 4:** Activation parameters of 304L stainless steel corrosion in physiological serum, both in the absence and presence of different concentrations of HKER.

Sample	*E_a_*/kJ mol^−1^	ln*A* = y (x = 0)	*A*/g m^−2^ h^−1^ A = e^y(x=0)^	Δ*H_a_*/kJ mol^−1^	*E_a_ −* Δ*H_a_*	Δ*S_a_*/J mol^−1^ K^−1^
SS/PS	24.44	8.3547	4250.1	21.91	2.53	−183.91
SS/PS/10 mg L^−1^ HKER	24.37	8.1884	3598.9	-	-	-
SS/PS/15 mg L^−1^ HKER	24.31	7.6599	2121.5	-	-	-
SS/PS/20 mg L^−1^ HKER	24.26	7.7766	2384.2	-	-	-
SS/PS/25 mg L^−1^ HKER	27.62	8.8215	6778.4	24.97	2.65	−180.34
SS/PS/30 mg L^−1^ HKER	38.53	12.720	334,368.8	36.07	2.46	−147.34
SS/PS/35 mg L^−1^ HKER	40.80	13.276	583,033.5	38.21	2.59	−143.15
SS/PS/40 mg L^−1^ HKER	41.84	13.212	546,888.4	39.35	2.49	−143.33

**Table 5:** The degree of surface coverage (*θ*) of 304L stainless steel by HKER adsorption, at different temperatures.

Sample	Surface Coverage Degree (*θ*)			
	25 °C	35 °C	45 °C	55 °C
SS/PS	-	-	-	-
SS/PS/10 mg L^−1^ HKER	0.143	0.126	0.116	0.140
SS/PS/15 mg L^−1^ HKER	0.270	0.255	0.318	0.269
SS/PS/20 mg L^−1^ HKER	0.388	0.429	0.374	0.404
SS/PS/25 mg L^−1^ HKER	0.557	0.539	0.497	0.488
SS/PS/30 mg L^−1^ HKER	0.725	0.685	0.588	0.566
SS/PS/35 mg L^−1^ HKER	0.806	0.745	0.701	0.660
SS/PS/40 mg L^−1^ HKER	0.886	0.862	0.807	0.758

**Table 6:** Adsorption parameters of HKER on 304L stainless steel, obtained from Temkin isotherm and Awady’s model in physiological serum in various conditions, at different temperatures.

*T*/K	From Temkin Adsorption Isotherm							
	*f*	R^2^	*K_ads_*/ L g^−1^	$\Delta G_{ads}^{o}$/ kJ mol^−1^	$\Delta H_{ads}^{o}$/kJ mol^−1^		$\Delta S_{ads}^{o}$/J mol^−1^K^−1^	
					From Figure 10a	From Figure 10b	From Figure 10a	From Figure 10b
298	1.53	0.988	97.51	−28.5	−6.34	−6.726	74.2	73
308	1.45	0.9792	89.12	−29.2	−6.34	−6.726	74.2	73
318	1.5	0.9904	82.27	−29.9	−6.34	−6.726	74.2	73
328	1.51	0.9996	77.48	−30.7	−6.34	−6.726	74.2	73
***T*** **/K**	**From Awady’s Adsorption Model**							
	***y***	**R^2^**	***K_ads_*** **/** **L g^−1^**	$\Delta G_{ads}^{o}$ **/** **kJ mol^−1^**	$\Delta H_{ads}^{o}$ **/kJ mol^−1^**		$\Delta S_{ads}^{o}$ **/J mol^−1^K^−1^**	
					**From Figure 10a**	**From Figure 10b**	**From Figure 10a**	**From Figure 10b**
298	3.38	0.9986	43.71	−26.5	−5.2	−4.726	71.4	73
308	3.63	0.9995	41.71	−27.2	−5.2	−4.726	71.4	73
318	3.39	0.9889	37.57	−27.9	−5.2	−4.726	71.4	73
328	2.93	0.996	36.58	−28.7	−5.2	−4.726	71.4	73

**Table 7:** AFM parameters obtained for steel corroded in PS blank solution and in PS containing HKER at 25 °C.

Sample	*R_p−v_*/nm	*R_q_*/nm	*R_a_*/nm
SS standard	845	125	90
SS/PS blank	1318	281	198
SS/PS/30 mg L^−1^ KERH	1047	156	117
SS/PS/40 mg L^−1^ KERH	948	149	108

## Data Availability

Data are contained within the article.
